# Synergistic effects of Cu-doped ZnO nanoantibiotic against Gram-positive bacterial strains

**DOI:** 10.1371/journal.pone.0251082

**Published:** 2021-05-14

**Authors:** Awais Khalid, Pervaiz Ahmad, Abdulrahman I. Alharthi, Saleh Muhammad, Mayeen Uddin Khandaker, Mohammad Rashed Iqbal Faruque, Israf Ud Din, Mshari A. Alotaibi, Abdulhameed Khan

**Affiliations:** 1 Department of Physics, Hazara University, Mansehra, Pakistan; 2 Department of Physics, Faculty of Science, University of Malaya, Kuala Lumpur, Malaysia; 3 Department of Physics, University of Azad Jammu and Kashmir, Muzaffarabad, Pakistan; 4 Department of Chemistry, College of Science and Humanities, Prince Sattam Bin Abdulaziz University, Al-Kharj, Saudi Arabia; 5 Center for Applied Physics and Radiation Technologies, School of Engineering and Technology, Sunway University, Selangor, Malaysia; 6 Space Science Centre, Universiti Kebangsaan Malaysia (UKM), Selangor, Malaysia; 7 Department of Biotechnology, University of Azad Jammu and Kashmir, Muzaffarabad, Pakistan; College of Engineering, University of Saskatchewan, CANADA

## Abstract

A viable hydrothermal technique has been explored for the synthesis of copper doped Zinc oxide nanoparticles (Cu-doped ZnO-NPs) based on the precursor’s mixture of Copper-II chloride dihydrate (CuCl_2_.2H_2_O), Zinc chloride (ZnCl_2_), and potassium hydroxide (KOH). X-ray diffraction (XRD) reported the hexagonal wurtzite structure of the synthesized Cu-doped ZnO-NPs. The surface morphology is checked via field emission scanning electron microscopy (FE-SEM), whereas, the elemental compositions of the samples were confirmed by Raman, and X-ray photoelectron spectroscopy (XPS), respectively. The as-obtained ZnO-NPs and Cu-doped ZnO-NPs were then tested for their antibacterial activity against clinical isolates of Gram-positive (*Staphylococcus aureus*, *Streptococcus pyogenes*) and Gram-negative (*Escherichia coli*, *Klebsiella pneumonia*) bacteria via agar well diffusion method. The zone of inhibition (ZOI) for Cu-doped ZnO-NPs was found to be 24 and 19 mm against S. Aureus and S. pyogenes, and 18 and 11 mm against E. coli and K. pneumoniae, respectively. The synthesized Cu-doped ZnO-NPs can thus be found as a potential nano antibiotic against Gram-positive multi-drug resistant bacterial strains.

## 1. Introduction

Zinc oxide nanoparticles (ZnO-NPs) are the most promising metal oxides semiconductor with a high exciton binding energy (60 MeV) and a direct band gap of 3.37 eV [1]. It is the most widely used nanomaterial as UV absorbers in textiles [2], hybrid solar cells [3], varistor fabrication [4], light-emitting diodes (LEDs) [5], wastewater treatment applications [6], and emission control [7]. ZnO-NPs has excellent biomedical properties to be effectively utilized as diagnosis, antimicrobial agent, bio-imaging, drug delivery, and in cancer treatment, etc. [8–10]. Compared to normal cells, ZnO-NPs exhibit a favorable capacity to destroy human cancer cells [11] and can therefore be a potential candidate for anticancer activities [12, 13]. The potential cytotoxicity function of ZnO-NPs has been related to apoptosis occurrence [14]. Moreover, new approaches are needed for biomedical applications of ZnO-NPs to actively perform in antimicrobial and antibacterial activities [13, 15]. For this to happen, the properties and functionality of ZnO-NPs were needed to improve by incorporating other dopants materials that were sought to be the transition metals (Fe, Mn, Cu, Cr) or biomolecules at the nanoscale [16, 17]. After doping with transition metals and biomolecules, the surfaces of these nanoparticles are modified to have excellent biocompatibility to effectively perform in antimicrobials, antioxidants, drug delivery systems, bio-imaging, and biosensors, etc. [18]. Among all other dopants, Cu^2+^ is found to significantly change the morphological, structural, optical, electrical, magnetic, and biological properties of the ZnO-NPs. Because of biomedical application, Cu^2+^ is considered to be the best option [19], because, it has a comparable similar size of ionic radii to that of Zn^2+^ (0.73 Å, and 0.74 Å) and non-toxic to biological species. The combined antibacterial properties of CuO and ZnO can be investigated by doping Cu into a ZnO matrix or Zn into a CuO matrix [20]. Besides, doping acquires the dopant to have excellent electrical conductivity. Moreover, it can easily modify or change the chemical and physical properties of ZnO [21, 22]. Similarly, Cu-doped ZnO-NPs show an increase in energy conversion efficiency and storage capacity of electrochemical cells [23].

Because of the significant improvement in the properties of ZnO-NPs with Cu-doping, several techniques have been reported in the past [24]. Cu-doped ZnO-NPs has been reported with a band gap of ~3.4 eV by the co-precipitation method. It was found that the copper concentration as dopants has a key role in the improvement of photoluminescence properties [25]. Likewise, ZnO and Cu-doped ZnO nanorods were reported by the mechanically assisted thermal decomposition process. Cu-doped ZnO nanorods were found to exhibit better photocatalytic and antibacterial characteristics than pure ZnO nanorods [26]. Several more techniques have been reported for the synthesis of Cu-doped ZnO-NPs including sol-gel, co-precipitation, sonochemical synthesis, vapor transport technique, hydrothermal, and combustion method, etc. [27, 28]. In most of the reported techniques, the Cu contents were not sufficient to tailor the inherited properties of ZnO for the targeted applications. Besides, most of the techniques were complex, lengthy, and found to contain some of the precursors as impurities in the final product. Unlike all the above, a simple and practical hydrothermal technique has been explored based on the precursor’s mixture of CuCl_2_. 2H_2_O, ZnCl_2,_ and KOH. The precursors’ types, their ratios, and catalyst are carefully selected by keeping in mind the content of the final product. Catalytically the precursors are allowed to react (after their initial decomposition) and form bulk product (ZnO) along with a metallic catalyst (Cu). The bulk ZnO is reduced to the lower dimension (Nanoscale) and then Cu-doped by the dual activity of Copper (released from CuCl_2_. 2H_2_O).

It has already been stated that doping is the most extensively studied method for the modification of nanoparticles to enhance their (biomedical) properties [29]. Moreover, it has been shown that metal-doped ZnO nanoparticles have more antibacterial activity than undoped ZnO nanoparticles against both Gram-negative and Gram-positive bacteria [30, 31]. In this regard, the antibacterial potential of the synthesized Cu-doped ZnO-NPs is also evaluated by the agar well diffusion method [32] against clinical isolates of both gram-positive and gram-negative bacteria.

## 2. Experimental details

### 2.1 Materials

Zinc chloride (ZnCl_2_), Copper II chloride dihydrate (CuCl_2_∙2H_2_O), and Potassium hydroxide (KOH) were purchased from Sigma Aldrich. Four different bacterial strains (obtained from Combined Military Hospital (CMH) Muzaffarabad) including, two gram-positive bacteria: *Streptococcus pyogenes* (*S*. *pyogenes*; ATCC^®^ 25063) and *Staphylococcus aureus* (*S*. *aureus*; ATCC^®^ 25923) and two gram-negative bacteria: *Escherichia coli* (*E*. *coli*; ATCC^®^ 25922) and *Klebsiella pneumonia* (*K*. *pneumonia*; ATCC^®^ BAA-1144) was used to perform the antibacterial activity. Mueller-Hinton Agar (MHA) powder and nutrient broth powder was purchased from Sigma-Aldrich.

### 2.2 Synthesis and Cu-doping of ZnO

Copper-II chloride dihydrate (CuCl_2_. 2H_2_O), Zinc chloride (ZnCl_2_), and potassium hydroxide (KOH) are taken as precursors. At first, a mixture (in a ratio of 1: 3) of 0.5 g of CuCl_2_. 2H_2_O and 1.5 g of ZnCl_2_ is dissolved into 50 ml of deionized water. Afterward, 1g of KOH is separately dissolved in 20 ml of deionized water and added dropwise to the already made homogeneous mixture solution of CuCl_2_.2H_2_O and ZnCl_2_. The solution mixture is then stirred at room temperature for 30 min. Consequently, a few drops of ethanol are also added to the solution mixture. The solution mixture is then taken in a Teflon-lined autoclave and placed inside an oven at 170°C for 22 h. As a result, a white precipitate has been obtained. The as-obtained white precipitate is then washed several times with double distilled water and ethanol. Finally, the precipitate is kept for drying in the furnace at 130°C for 1.5 hours.

### 2.3 Characterization tools

The surface morphology and shape of the as-synthesized Cu doped ZnO-NPs were examined via FE-SEM (JSM 7600F, JEOL, Japan). The composition and phase of Cu doped ZnO samples were investigated by XRD (PXRD, Shimadzu X-600, Japan with CuK radiation of λ = 1.54056 Å) and XPS. The Raman spectra of the samples were recorded (in the spectral range of 200–600 (cm ^-1^)) with a Dispersive Micro Raman system (In via, Renishaw), equipped with 514 nm diode-laser.

### 2.4 Screening of antibacterial activity of Copper doped ZnO-NPs

For screening of antibacterial activity of Cu-doped ZnO-NPs, all bacterial strains are sub-cultured from their pure cultures in Mueller–Hinton broth media and subjected to overnight incubation at 37°C. The turbidity of bacterial culture is adjusted to freshly prepared 0.5 McFarland turbidity standard [33] equivalents to (1.5×10^8^ CFU/mL) bacteria. Each bacterial species is swabbed aseptically onto separate Muller–Hinton agar plates with the help of sterile cotton swabs. Wells are made with a sterile polystyrene tip (4 mm). Different concentrations of Cu-doped ZnO-NPs (0.1, 0.5 and 1 mg / mL) are prepared separately in 2% Hydrochloric acid (HCl) and used. Forty microliters (40 μL) of each concentration is then added to each well. All the plates are incubated overnight at 37°C. The zone of inhibition (ZOI) around each well is measured in millimeters by using a caliper. Clindamycin phosphate at a concentration of 20 μg/mL is used as a standard reference antibiotic. Each experiment is performed in triplicate (N = 3) and the mean value is calculated.

## 3. Results and discussion

Catalysts and their catalytic activities are playing key roles in the synthesis of both bulk and nanomaterials or their doping (for changing or modifying their properties) via a range of techniques. One such technique has been designed and operated for the synthesis of Cu-doping of ZnO. Under the catalytic activity of KOH, CuCl_2_. 2H_2_O and ZnCl_2_ are allowed to react. The reaction thus happened to forms bulk ZnO as well as Cu. Cu is further planned to work in two ways. At first, Cu acts as a catalyst to work in the size reduction of ZnO from bulk to nano. Afterward, Cu adjusts itself in the lattice sites of ZnO as a dopant to form Cu-doped ZnO.

### 3.1 Morphology

**Fig 1:**
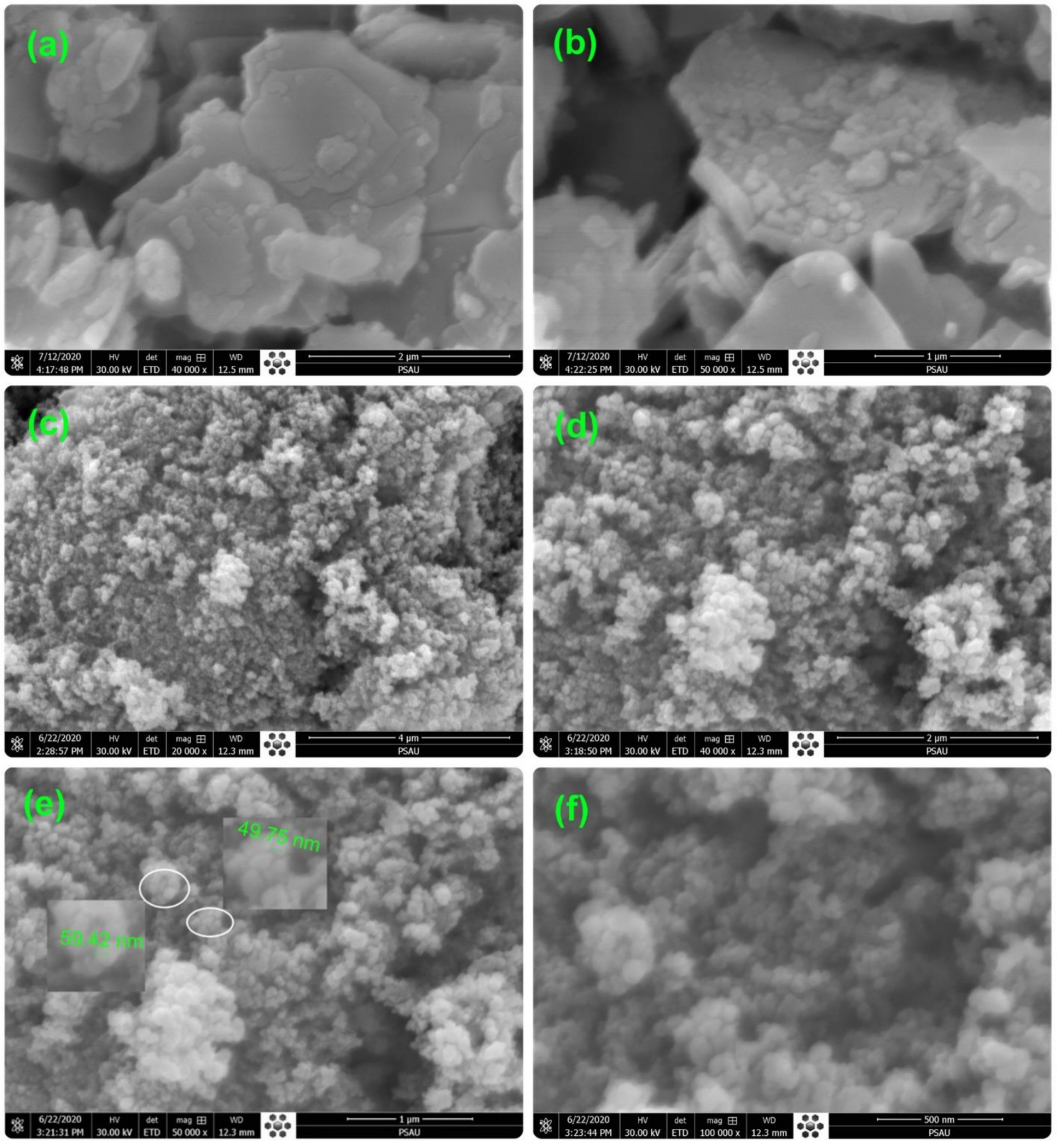
FESEM micrograph of pure and Cu-doped ZnO-NPs: pure ZnO (a) 2 μm and (b) 1 μm, Cu doped ZnO (c) 4 μm (d) 2 μm (e) 1 μm (f) 500 nm.

Hydrothermally synthesized ZnO and Cu-doped ZnO-NPs are first characterized via FE-SEM to observe its apparent morphology. **Fig 1** shows the structure and morphology of the synthesized ZnO and Cu-doped ZnO-NPs in (a) low (40,000 x) (b) high (50,000 x) magnified ZnO and (c) low (20, 000 x), (d) high (40,000 x), (e) higher (50,000 x), and (f) highest magnification (100000 x) Cu-doped ZnO-NPs. The morphology of ZnO seems like to be nanosheets in both Fig 1(A) and 1(B) micrographs, which were trimmed due to addition of Cu. All the Cu-doped ZnO-NPs seem like smaller size isolated cotton packs placed along, side by side at the bottom. Most of the smaller size particles are closely packed alongside others at the bottom, as can be seen in Fig 1(C). Fig 1(D) shows that the larger size particles are placed at the top and mostly agglomerated. The agglomerated packs virtually seem to be the as-synthesized bulk ZnO formed by the initial reactions of the precursors. Each agglomerated pack of bulk ZnO is a collection of several nano-sized particles. These particles might have been reduced to the nanoscale by the catalytic reaction of Cu. A few of these agglomerated packs are encircled white and further magnified in Fig 1(E) with their calculated size. Nanoparticles in a bit larger circle are magnified shown on the left (with calculated and denoted size of 59.42 nm). Similarly, the nanoparticles encircled in a bit smaller circle is magnified on the right with a measured size of 49.75 nm. The observation of the FESEM results in Fig 1(F) shows the variation in nanoparticle size and morphology within a dimension of below 100 nm. The size and shape of ZnO nanoparticles are found to depend on the Cu additive. These findings are found to be in close agreement with the previous findings [34].

### 3.2 Compositions and phase

**Fig 2:**
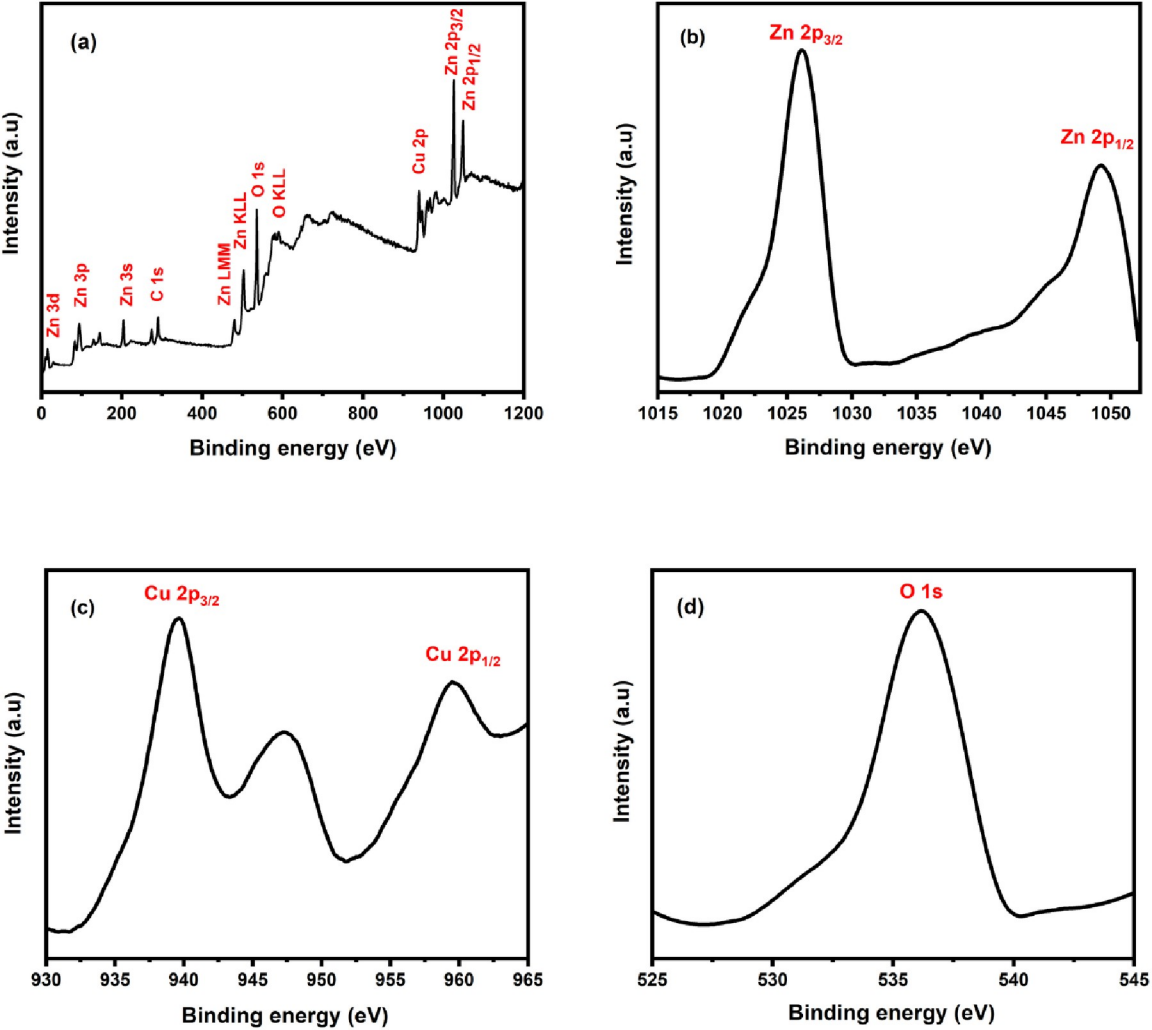
(a) XPS survey of the as-synthesized Cu-doped ZnO nanoparticles. (b) High resolution Zn 2p, (c) Cu 2p, and (d) O 1s XPS spectra.

**Fig 2** shows the XPS results for the chemical compositions, valence states, and bonding properties of the synthesized Cu-doped ZnO-NPs. In the XPS analysis, Zn 2p, Cu 2p, and O 1s are observed. No other magnetic contaminants or precursor elements are found in the sample. Besides, the C 1s peak is detected at 285 eV, which might be due to hydrocarbon contamination [35]. The XPS survey with the peaks tagged for the corresponding elements for their binding energies is shown in **Fig 2(A).** The survey shows not only the strong intensities of O 1s and Zn 2p but also the Cu 2p peaks for the Cu-contents. In **Fig 2(B)** two sharp peaks are observed corresponded to the spin-orbit of Zn 2p_3/2_ and Zn 2p_1/2_ at binding energies 1022.1 eV and 1045.2 eV respectively. No obvious peak shift is observed. The values match with that of the standard ZnO sample. This shows that Zn is present in the +2-oxidation state in the ZnO lattice. **Fig 2(C)** shows the Gaussian-fitted high-resolution XPS scans. Cu core level splits to Cu 2p_3/2_ and Cu 2p_1/2_ at binding energies of ~ 934.1 to 954.3 eV respectively. These are in good agreement with the previously reported values, thereby indicating that Cu has a divalent valence state [36]. Besides, the satellite peak attributed to the electron shake-up of cupric oxide (Cu^2+^) is found in the range of 940–945 eV [37]. This indicates that Cu ions were oxidized (Cu^2+^) in the ZnO nanoparticles and were substituted into the ZnO lattice at the Zn^2+^ site. **Fig 2(D)** shows the magnified O 1 s peak. The broad peak is assigned to O^2−^ ions at a binding energy of 530.9 eV in the Zn–O bonding of the wurtzite structure of ZnO. The convoluted peak at 532.6 eV is attributed to O^−^ and O^2−^ ions in oxygen-deficient regions in the sample matrix [38].

**Fig 3:**
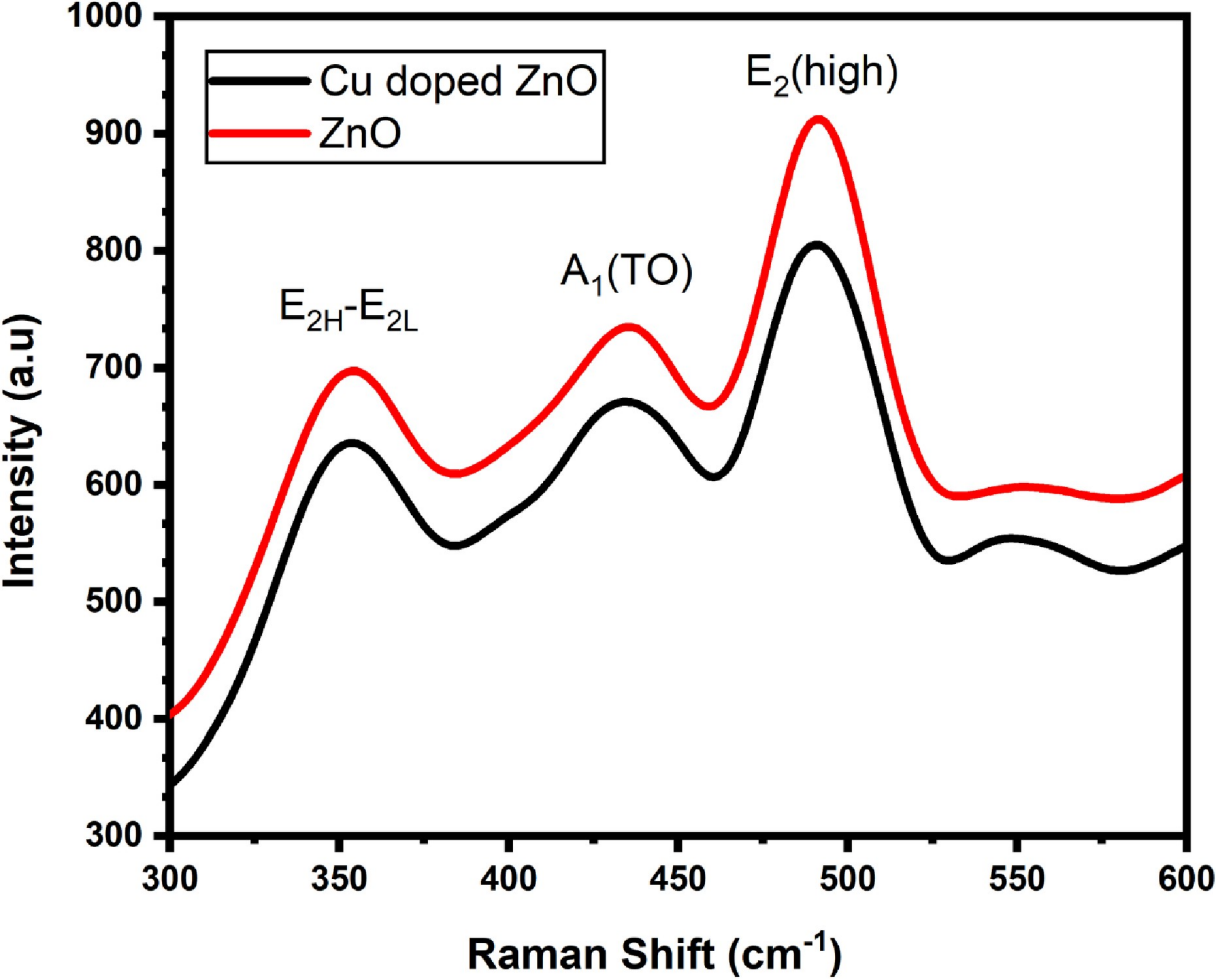
Raman spectrum of the as-synthesized ZnO and Cu-doped ZnO nanoparticles.

The measurements of Raman spectroscopy are performed in the spectral range of 200–600 (cm ^-1^). The reported Raman spectrum in **Fig 3** shows various peaks for pure and Cu doped ZnO-NPs located at 355 (cm^-1^), 434 (cm^-1^) and 493 (cm^-1^) respectively. These peaks are attributed to E_2H_ –E_2L_, A_1_ transverse optical (TO), and E_2_ (high) mode respectively [39]. The results according to the literature showed that when the Cu doping concentration is increased, the intensities of the peaks in the spectrum decrease, and the A_1_ (TO) phonon mode vanishes [40]. According to group theory, the other reported peaks in the Raman spectrum corresponds to different optical modes. Here, A_1_ and E_1_ modes, are polarized and can be divided into transverse optical (TO) and longitudinal optical (LO) phonons [41].

**Fig 4:**
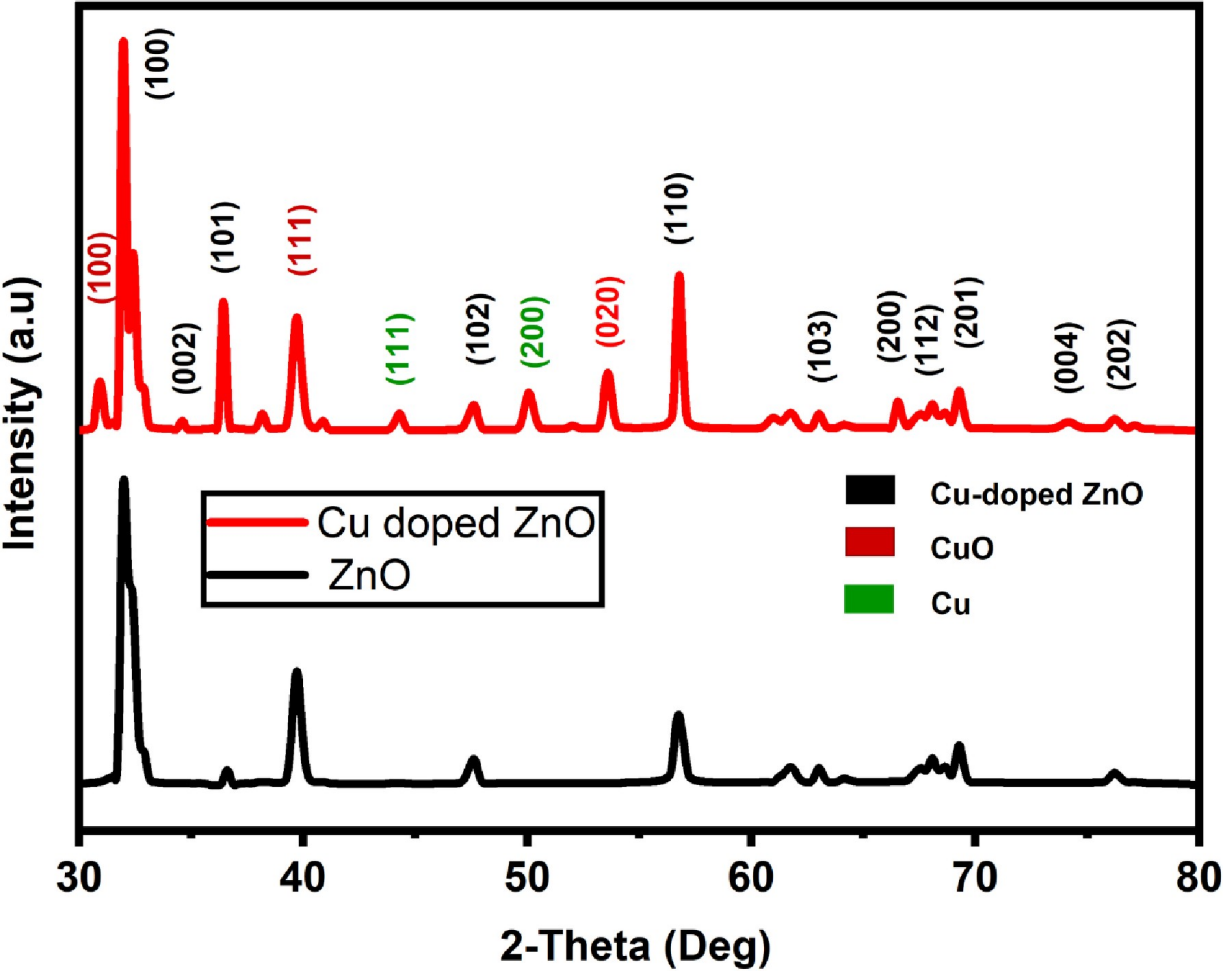
XRD pattern of Cu-doped ZnO nanoparticles showing peaks for different contents in the sample.

**Fig 4** shows the observed X-ray diffraction pattern of ZnO and Cu-doped ZnO. The crystallite size, phase, and structure of the material are determined with the help of positions and intensities of the peaks. The peaks in the XRD pattern appear at 31.9°, 34.4°, 36.5°, 47.5°, 56.7°, 62.8°, 66.5°, 67.8°, and 69.1° corresponds to (100), (002), (101), (102), (110), (103), (200), (112), (201), (004), and (202) planes in hexagonal wurtzite ZnO and Cu-doped ZnO respectively (JCPDS card No. 89–7102) [42, 43]. The XRD pattern also has several more peaks located at 30.9°, 38.8°, 44.5°, 50.2°, and 53.5°. The peaks at 30.9°, 38.8°, and 53.5° correspond to (100), (111), and (020) in Cu (II) oxide, whereas, the peaks at 44.5°, and 50.2° are assigned to the FCC phase of Copper [44]. The existence of Cu and CuO indicates the Cu-doping of the as-synthesized ZnO.

### 3.3 Antibacterial activity of Cu-doped ZnO-NPs

The antibacterial potential of synthesized copper doped ZnO-NPs is evaluated by the agar well diffusion method [32] against clinical isolates of both gram-positive and gram-negative bacteria obtained from Combined Military Hospital (CMH) Muzaffarabad. Total of 4 bacterial strains i.e. *Escherichia coli* (*E*. *coli* isolated from UTI), *Klebsiella*. *pneumonia* (K. *pneumonia* isolated from Wound) as Gram-negative and *Staphylococcus aureus* (S. *aureus* isolated from UTI), *Streptococcus pyogenes (S*. *pyogenes* isolated from throat swab*)* as Gram-positive bacteria are used in this study. All bacterial strains are identified by various biochemical tests according to the method described [45]. Pure culture of all bacteria is kept at 4°C in agar slants in a freeze-dried condition until later use.

**Fig 5:**
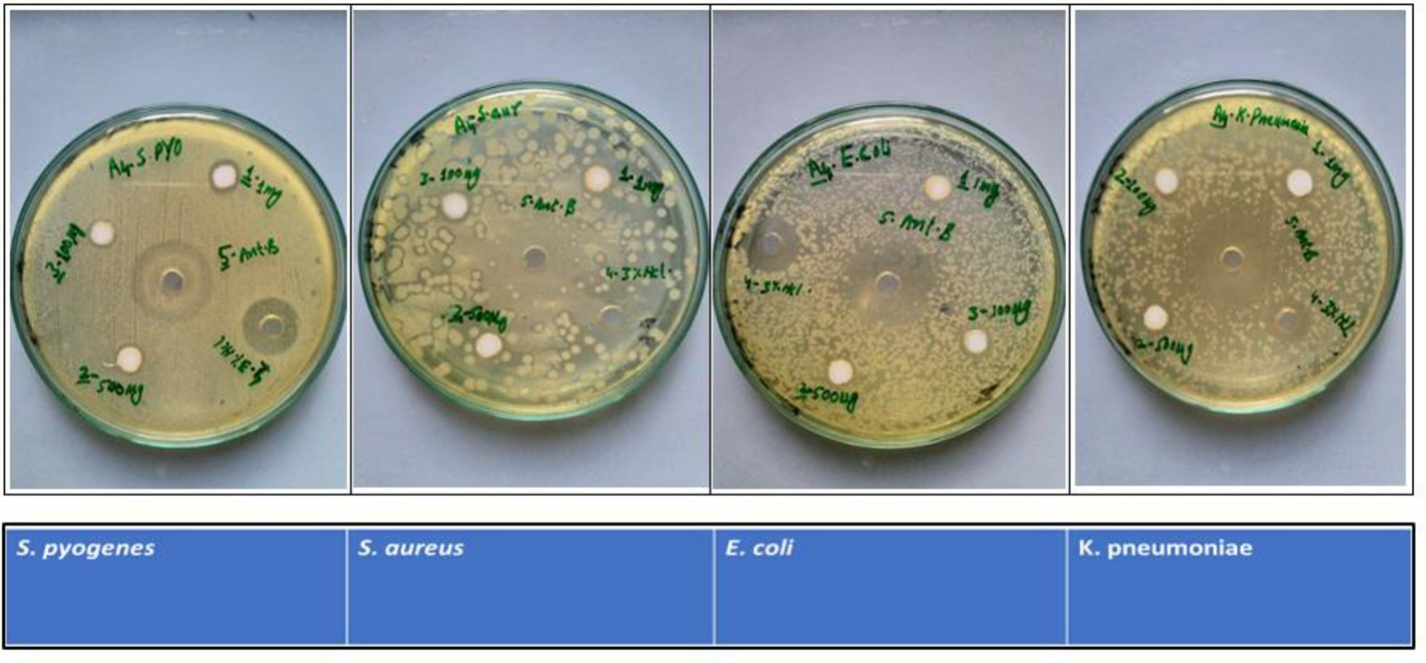
Zone of inhibition formed by ZnO-NPs against different bacteria.

**Fig 6:**
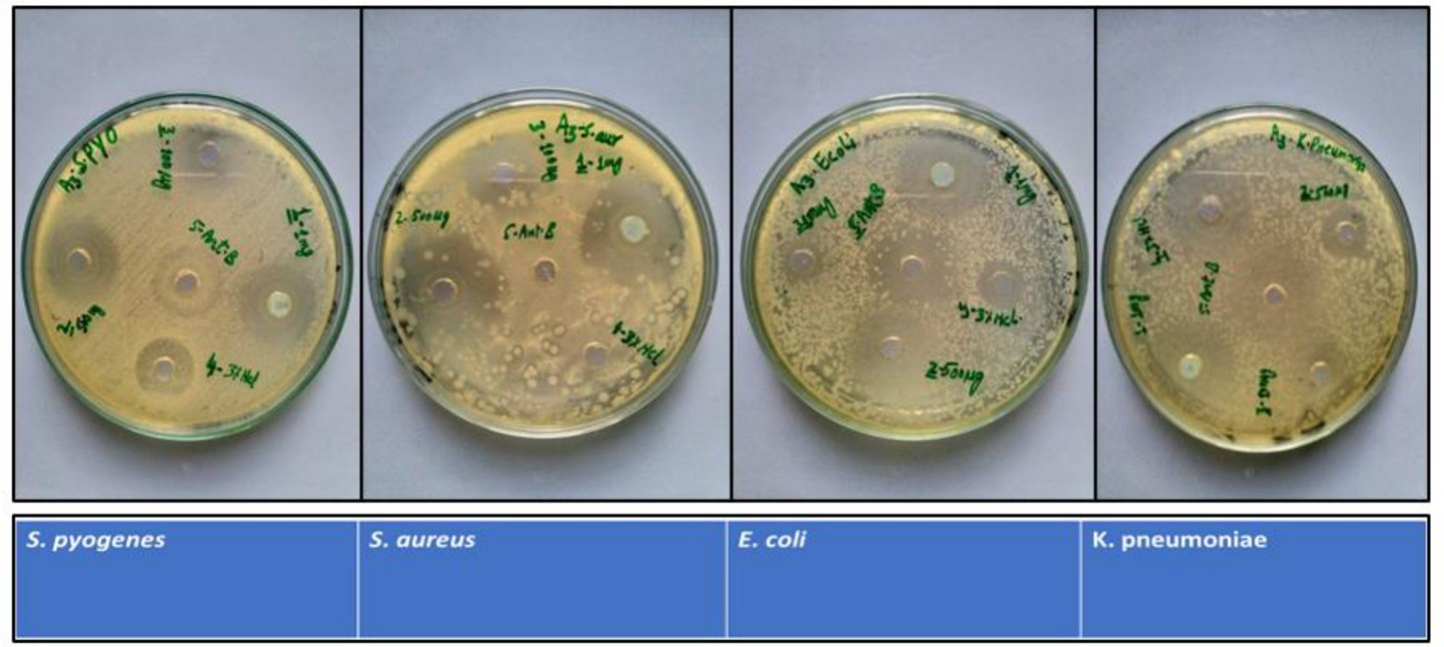
Zone of inhibition formed by Cu-doped ZnO-NPs against different bacteria.

The antibacterial effects of the Cu-doped ZnO-NPs are tested against four bacterial strains, of which two are gram-positive (*S*. *aureus*, *S*. *pyogenes*) and two are gram-negative (*E*. *coli*, *K*. *pneumonia*). The bacterial cultures are treated with various doses of Zn-NPs and Cu-doped ZnO-NPs (0.1, 0.5 and 1mg/mL) dissolved in 2% HCl. The results demonstrate that Cu-doped ZnO-NPs inhibit the growth of all the tested microbes more than ZnO-NPs in all the tested doses, as shown in **Figs 5 and 6**. The ZOI increases with an increase in the concentration of Cu-doped ZnO-NPs. We observed that Gram-positive microbes are more susceptible to Cu-doped ZnO-NPs as compared to Gram-negative microbes. For ZnO-NPs; gram-positive microbes, *S*. *aureu*s forms a ZOI of 13 ± 0.26 mm, whereas *S*. *pyogenes* form a ZOI of 9 ± 0.18 mm and gram-negative microbes, *E*. *coli* display 14 ± 0.28 mm ZOI, whereas *K*. *pneumonia* forms 15 ± 0.3 mm ZOI on the same dose. For Cu-doped ZnO-NPs, among gram-positive microbes, *S*. *aureu*s forms a ZOI of 24 ± 0.20 mm, whereas *S*. *pyogenes* form a ZOI of 20 ± 0.14 mm on the same dose. Among gram-negative microbes, *E*. *coli* display 18 ± 0.11 mm ZOI, whereas, the *K*. *pneumonia* forms 17 ± 0.13 mm ZOI as shown in **Table 1**. All the results were carried out in triplicate and mean diameter of inhibition zone was recorded and evaluated by using SPSS version 25.

**Table 1:** Zone of inhibition values (mm) of different concentrations of ZnO-NPs and Cu-doped ZnO-NPs against different bacteria.

Bacteria			ZnO			Cu doped ZnO		
			100 μg/ml	500 μg/ml	1 mg/ml	100 μg/ml	500 μg/ml	1 mg/ml
Gram-negative	*E*. *Coli*	Inhibition zone (mm)	11 ± 0.22	13 ± 0.26	14 ± 0.28	10 ± 0.2	15 ± 0.3	18 ± 0.36
	*K*. *Pneumoniae*		10 ± 0.2	11 ± 0.22	15 ± 0.3	09 ± 0.18	13 ± 0.26	17 ± 0.34
Gram-positive	*S*. *aureus*		10 ± 0.2	12 ± 0.24	13 ± 0.26	17 ± 0.34	23 ± 0.46	24 ± 0.48
	*S*. *pyogenes*		8 ± 0.16	9 ± 0.18	9 ± 0.18	12 ± 0.24	19 ± 0.38	20 ± 0.4

**Fig 7:**
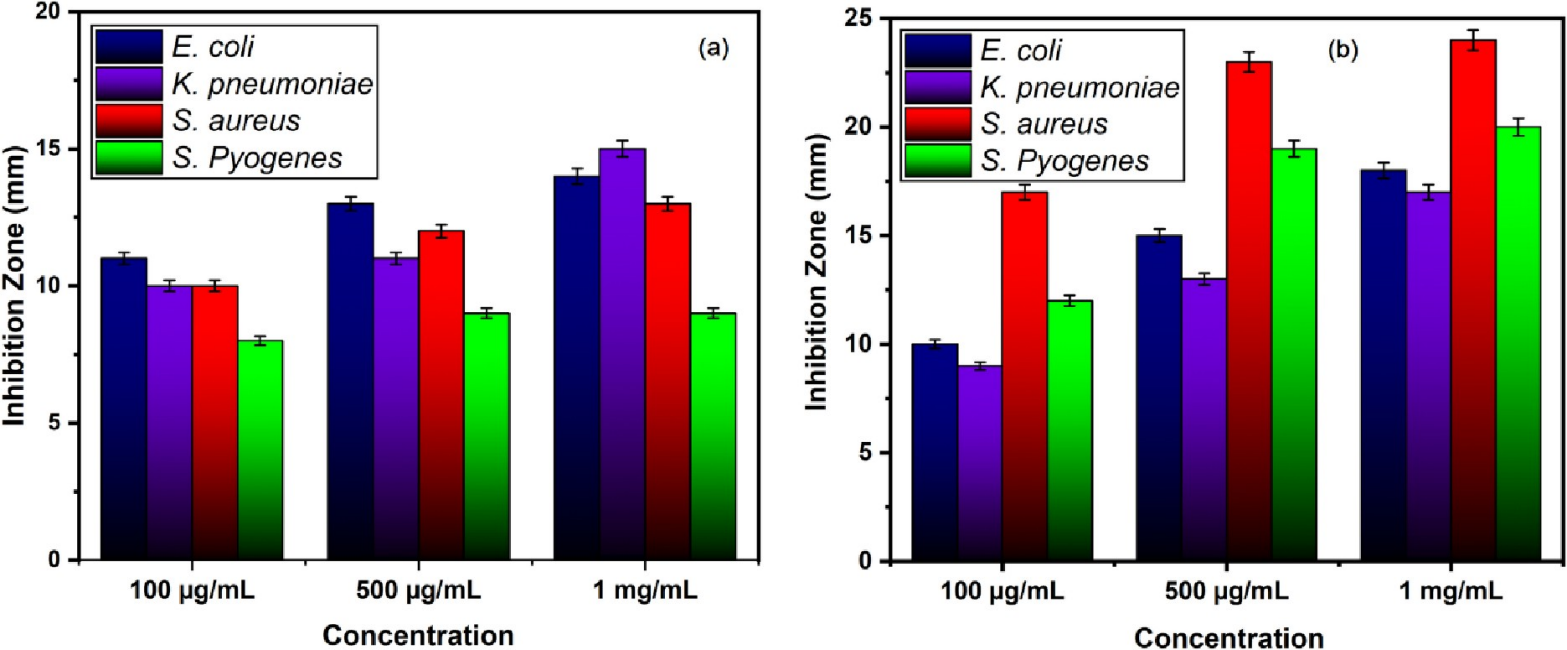
Bar graph showing the diameter of the zone of inhibition (in mm) produced by (a) ZnO-NPs and (b) Cu doped ZnO-NPs against gram positive and gram-negative bacteria.

**Fig 8:**
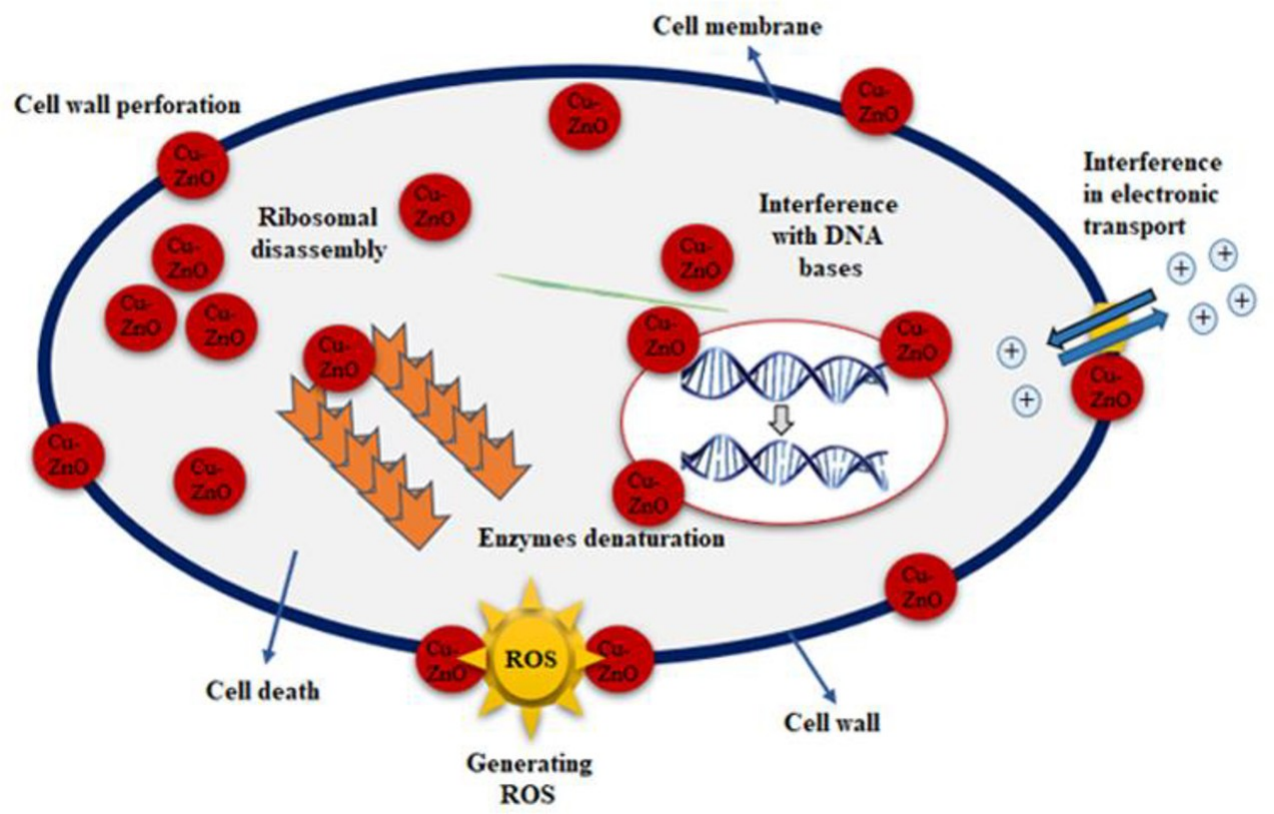
Schematic antimicrobial mechanism of Cu-doped ZnO-NPs against microbial strain.

Our findings are supported by several studies indicating that Cu-doped ZnO-NPs reported in the current work exhibit better antibacterial activity (than pure ZnO) towards Gram-positive bacteria than towards Gram-negative bacteria [46, 47]. The difference in antibacterial activity of ZnO-NPs and Cu-doped ZnO-NPs was clearly seen in **Fig 7**. This difference may be either due to structural differences among Gram-positive and Gram-negative bacteria or due to increase doping concentration of Copper. Gram-negative bacteria have an outer membrane and may reduce the damage from Cu-doped ZnO-NPs [48]. This has been supported by the findings that the Cu-doped ZnO-NPs or powders in an aqueous solution can produce various reactive oxygen species (ROS) such as hydroxyl radicals (OH), singlet oxygen, or superoxide anion (O^2−^) [49]. Hydroxyl radicals and singlet oxygen species are negatively charged species that cannot penetrate the cell membrane [50]. Zn^2+^, Cu, Cu^1+^, and Cu^2+^ ions released from Cu-doped ZnO-NPs are cytotoxic to microbes [51]. It is well known that zinc ions in high concentrations can negatively influence multiple activities in bacteria, such as glycolysis, transmembrane proton translocation, and acid tolerance which can prolong the lag phase of bacteria [52]. Copper ions may bind with DNA molecules and disrupt the helical structure, resulting in cell death [53]. Another possible mechanism could be that Cu-doped ZnO induces the production of (ROS) via Fenton type reactions [51]. ROS induces oxidative stress which can impair bacterial membranes, lipids, protein, and DNA. Cytoplasmic contents are discharged as a result of ROS generation by NPS that mounts the microbial cell membrane. These ROS is responsible for the death of microbial strains as shown in **Fig 8**. The close association of cations (Cu^2+^ and Zn^2+)^ with negatively charged parts of the bacterial cell membrane is another potential mechanism for the reaction of nanomaterials to bacterial strains, resulting in the collapse of micro-pathogens [54–56]. However further studies are needed to explore the exact mechanism of Cu-doped ZnO-NPs antibacterial activity.

## 4. Conclusions

Potassium hydroxide is found to be an effective catalyst during the reaction of Copper-II chloride dihydrate and Zinc chloride. The liberated copper during the reaction is found to play an effective role in the size reduction of the as-synthesized ZnO and its Cu-doping. Cu-doped ZnO-NPs reported in the current work can effectively work in antibacterial activity against Gram-positive and Gram-negative bacteria. The results showed that Cu-doped ZnO-NPs exhibit better antibacterial activity towards Gram-positive bacteria than towards Gram-negative bacteria. Cu-doped ZnO-NPs was found to inhibit the growth of all tested bacteria. The inhibitory effect was enhanced in a dose-dependent manner. It has been observed that Gram-positive microbes are more susceptible to Cu-doped ZnO-NPs as compared to Gram-negative microbes. Cu-doped ZnO-NPs can further be investigated for antifungal, drug delivery, tissue engineering, and other biomedical applications.
